# Volumetric reference data of the orbit: a deep learning MRI analysis in the German national cohort

**DOI:** 10.1038/s41598-026-68393-x

**Published:** 2026-08-29

**Authors:** Navid Farassat, Marco Reisert, Susanne Rospleszcz, J. E. Rod, Daniel Böhringer, Thomas Kroencke, Thoralf Niendorf, Tobias Pischon, Henry Völzke, Steffen Ringhof, Thomas Reinhard, Fabian Bamberg, Wolf Alexander Lagrèze, Christopher L. Schlett, Kevin Wornath, Moises Felipe Molina-Fuentes

**Affiliations:** 1 Eye Center, Faculty of Medicine, Medical Center - University of Freiburg, University of Freiburg, Freiburg im Breisgau, Germany; 2 Medical Physics, Department of Diagnostic and Interventional Radiology, Faculty of Medicine, Medical Center - University of Freiburg, University of Freiburg, Freiburg im Breisgau, Germany; 3 Department of Diagnostic and Interventional Radiology, Faculty of Medicine, Medical Center - University of Freiburg, University of Freiburg, Freiburg im Breisgau, Germany; 4 Division of Health Sciences, Department of Public Health, Universidad del Norte, Barranquilla, Colombia; 5 Department of Diagnostic and Interventional Radiology, University Hospital Augsburg, Augsburg, Germany; 6 Centre for Advanced Analytics and Predictive Sciences, University of Augsburg, Augsburg, Germany; 7 Berlin Ultrahigh Field Facility (B.U.F.F.), Max Delbrueck Center for Molecular Medicine in the Helmholtz Association, Berlin, Germany; 8 Molecular Epidemiology Research Group, Max Delbrück Center for Molecular Medicine in the Helmholtz Association (MDC), Berlin, Germany; 9 Max Delbrück Center for Molecular Medicine in the Helmholtz Association (MDC), Biobank Technology Platform, Berlin, Germany; 10 Charité - Universitätsmedizin Berlin, Corporate Member of Freie Universität Berlin and Humboldt-Universität zu Berlin, Berlin, Germany; 11 Institute for Community Medicine, University Medicine Greifswald, Greifswald, Germany; 12 Department of Neuroradiology, Clinical Neuroscience Center, University Hospital Zurich, University of Zurich, Zurich, Switzerland

**Keywords:** Orbit, Morphometry, Reference data, NAKO, MRI, Germany, Anatomy, Computational biology and bioinformatics, Diseases, Health care, Medical research

## Abstract

Manual segmentation of orbital magnetic resonance imaging (MRI) is labor-intensive, hindering large-scale morphometric studies. To overcome this, we developed a fully automated deep learning pipeline to segment orbital MRIs and establish age- and sex-stratified normative reference data. We analyzed T1-weighted brain MRIs from 30,868 participants in the population-based German National Cohort (NAKO). After quality control, 28,779 participants (mean age 48.1 years; 44.1% female) were included. The model, validated against expert manual segmentations, accurately extracted 34 volumetric and geometric parameters across 15 orbital structures (Dice Similarity Coefficients: vitreous 0.97, lens 0.89, optic nerve 0.85). Mean [SD] axial length was 23.5 [1.2] mm. Mean [SD] volumes were 34.4 [3.9] cm³ for total orbital contents, 6.3 [0.8] cm³ for the vitreous, and 0.17 [0.03] cm³ for the lens. Males exhibited significantly larger dimensions across all parameters (*p* < 0.001). Age-stratified percentile curves revealed continuous age-dependent lens growth (Spearman’s ρ = 0.57 in men, 0.51 in women) alongside modest volume increases in the orbit, optic nerve, and extraocular muscles. This deep learning tool effectively resolved the bottleneck of manual segmentation, providing comprehensive orbital reference data for the German population. This foundation enables future high-throughput epidemiological research into the associations between orbital anatomy, systemic health, and disease.

**Supplementary Information:**

The online version contains supplementary material available at https://doi.org/10.1038/s41598-026-68393-x.

## Introduction

The human orbit is a complex anatomical region containing the eyeball and other structures essential for the visual system, including the optic nerve, extraocular muscles, and associated soft tissues. Their morphologies and volumes are pertinent to various physiological and pathological conditions^1,2^. For instance, orbital anatomy influences optic nerve mechanics, a factor implicated in optic neuropathies like glaucoma^3^, extraocular muscle (EOM) enlargement is a hallmark of thyroid eye disease^4–6^, and increased axial length leads to myopia^7^. Consequently, establishing robust reference data via precise orbital morphometry is crucial for clinical medicine, understanding pathophysiology, and identifying risk factors.

Magnetic resonance imaging (MRI) is the primary modality for visualizing orbital soft tissues, enabling the quantitative assessment of these structures^8^. However, this has traditionally relied on manual segmentation, where an expert delineates the boundaries of each structure. This approach is exceedingly labor-intensive, with a comprehensive segmentation of a single orbit often requiring several hours. This bottleneck renders quantitative analysis impractical for large populations. Furthermore, manual segmentation is susceptible to inter- and intra-observer variability, compromising reliability and reproducibility and hindering the detection of subtle morphological changes^9^.

Large population-based cohort studies like the German National Cohort (NAKO, *n* > 200,000 participants)^10,11^, provide an opportunity to study health and disease on a massive scale. With whole-body MRI data for over 30,000 participants, acquired using a strictly standardized imaging protocol across identical MRI scanners, NAKO provides a rich dataset for investigating links between eye and orbit morphology and a wide spectrum of demographic, anthropometric, and health-related parameters^12^. However, leveraging such vast datasets requires a shift away from manual analysis toward automated, high-throughput approaches.

Recent advancements in deep learning, particularly the success of convolutional neural networks in medical image analysis, have paved the way for such automated solutions^13,14^. These algorithms can be trained to perform complex segmentation tasks with an accuracy rivalling that of human experts while offering high consistency^15,16^. By applying these techniques to large cohorts, it becomes feasible to extract quantitative image-derived data efficiently and robustly, thereby unlocking new avenues for epidemiological research.

In this study, we capitalized on these developments to develop and validate a fully automated deep learning pipeline for the segmentation and morphometric analysis of the human eye and surrounding orbital tissues using T_1_-weighted MRI data from the NAKO cohort. Our objective was to establish comprehensive, age- and sex-stratified reference data for a wide range of orbital volumetric and geometric parameters in the German population, enabling future large-scale investigations into the links between orbital morphology, systemic health, and disease.

## Methods

### Study design and participants

This cross-sectional study analyzed baseline data from the prospective, population-based German National Cohort (NAKO) study. The NAKO study randomly recruited 205,415 participants (aged 20 to 69 years) from compulsory resident registries in the catchment areas of 18 study centers across Germany^11^.

A subsample of 30,868 participants (aged 20 to 74 years) underwent comprehensive, noncontrast whole-body MRI at five dedicated imaging centers between May 2014 and April 2019^12,17^.

For this study, we analyzed the complete NAKO MRI cohort with available orbital data. Demographic (age, self-reported sex) and anthropometric (e.g., height) data were assessed for each participant.

### Ethics

The NAKO study was conducted in accordance with the principles of the Declaration of Helsinki. Participants provided written informed consent. The protocol was approved by the NAKO ethics committee and the ethics committees of the participating study centers. This specific analysis was approved by the NAKO scientific and ethical advisory boards (project #NAKO-797). In addition, local Ethics Committee approval was obtained (#23-1316-S1-retro).

### MRI acquisition

Imaging was performed as described before^12,17–20^. In brief, the NAKO whole-body MRI protocol was performed on five identical 3-T scanners (MAGNETOM Skyra; Siemens Healthineers, Erlangen, Germany) using a standardized protocol.

For orbital morphometry, we utilized the 3-dimensional T_1_-weighted magnetization-prepared rapid gradient-echo (MPRAGE) sequence of the brain (isotropic spatial resolution = 1.0 × 1.0 × 1.0 mm³; matrix size = 256 × 256 pixels; 192 slices), acquired in sagittal orientation. The data were provided in the Neuroimaging Informatics Technology Initiative (NIfTI) format.

### Image segmentation

**Fig. 1:**
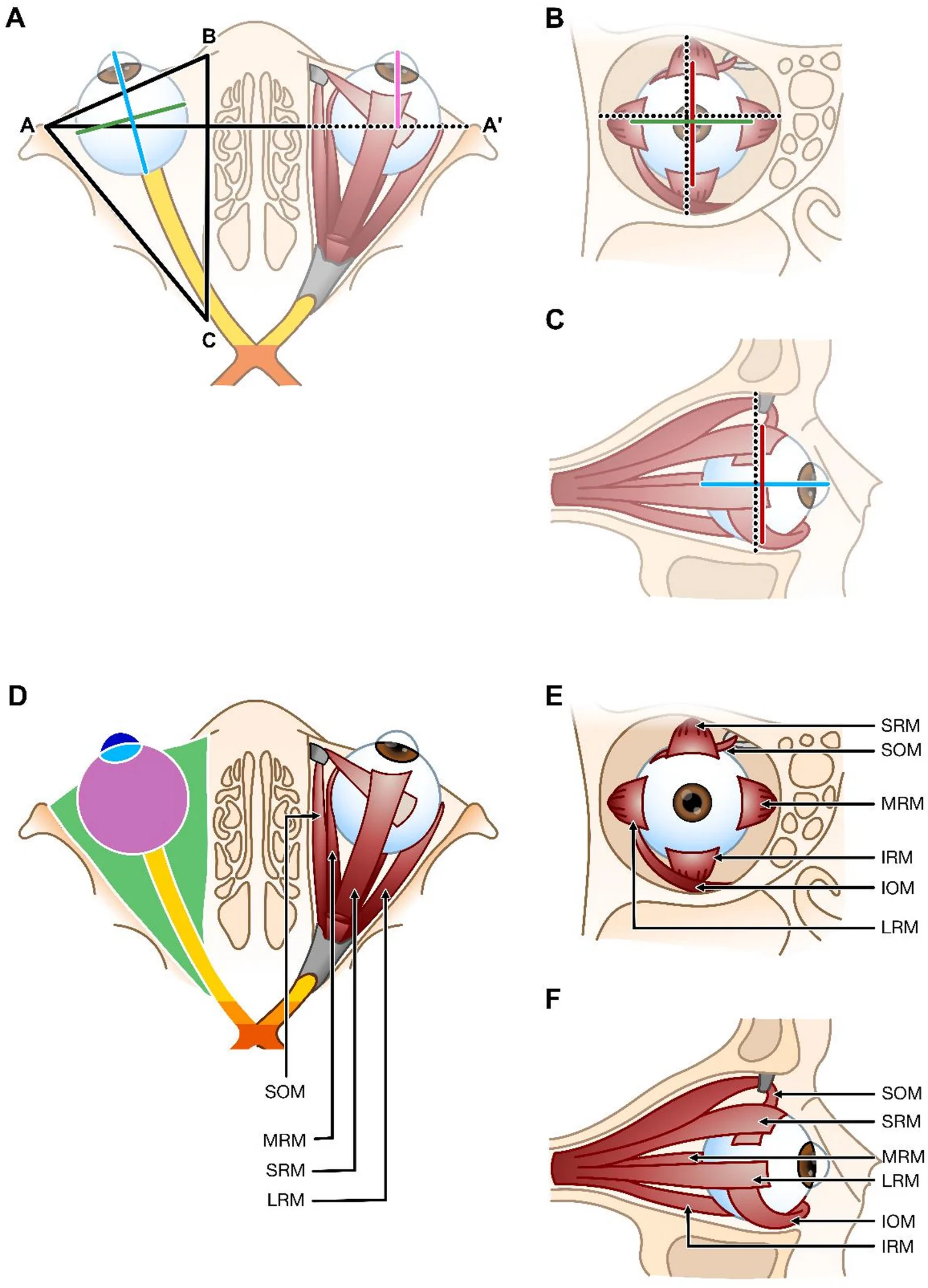
Target orbital morphometric parameters. Schematic representation of geometric parameters on T1-weighted MRI in transverse (**A**), coronal (**B**), and sagittal (**C**) planes. A/A‘: lateral orbital rim, B: medial orbital rim, C: orbital apex, AB: orbital opening, ABC°: orbital rim angle, A-> A‘: zygomatic line. Color key: : proptosis : horizontal eyeball diameter : vertical eyeball diameter : axial length. Dotted lines indicate the maximal vertical and horizontal orbital diameters. Segmented orbital structures as color overlays in transverse (D), coronal (E), and sagittal (F) planes of T1-weighted MRI images. Extraocular muscles (EOM): SRM: superior rectus muscle, IRM: inferior rectus muscle, MRM: medial rectus muscle, LRM: lateral rectus muscle, SOM: superior oblique muscle, IOM: inferior oblique muscle. Color key: : lens : anterior chamber : vitreous : optic nerve : optic chiasm : remaining non-bony orbital tissue.

All image processing, including segmentation, was performed using the Nora Medical Imaging platform (www.nora-imaging.com), an in-house developed framework for advanced medical image analysis^21^. We developed a fully automated deep learning pipeline to segment 15 orbital structures: anterior chamber, lens, vitreous, optic nerve (intraorbital and intracranial sections), optic chiasm, and the six extraocular muscles (medial, lateral, inferior, and superior rectus; inferior and superior oblique), as well as the complete nonbony orbit including lateral and medial orbital rims (Fig. 1).

#### Ground truth generation

Ground truth segmentations were generated via an iterative “expert-in-the-loop” process. Initial manual segmentations of 12 orbits served to train a prototype model; subsequent predictions on larger datasets were manually corrected by expert raters. A consensus-driven approach was used to resolve disagreements. This process was repeated until sufficient quality was reached, yielding a final training set of 322 orbits (161 participants).

#### Deep neural patchworks architecture

The model was trained using a Deep Neural Patchworks (DNP) segmentation framework based on the hierarchical and nested stacking of patch-based 3D networks of fixed matrix size (32 × 32 × 32) but decreasing physical input size. The size of the hierarchical pyramid (depth 4) was selected such that the field-of-view of each dimension covered 90% of the full acquisition window. The pyramid scaling was chosen so that the smallest layer reached an isotropic resolution of 1 mm. The architecture of the basis U-Net applied in this project closely followed the default U-Net configuration, with feature dimensions of 8, 16, 16, 32, and 64, and maximum pooling in the encoding layers and transposed convolutions in the decoding layers. The network was trained with the Adam optimizer (learning rate = 0.001). As a loss function, a categorical cross-entropy was used. The Patchwork CNN was trained for 15 million patches (batch size of 32), taking approximately two days on a GPU-accelerated server system with an RTX A6000 graphic unit (NVIDIA, Santa Clara, California, USA). During training, patches were randomly sampled so that approximately 50% of the finest patches contained at least one label. No systematic tuning was performed with the settings adapted to prior established values. The application of the trained network on a standard CPU system with 8 cores on an AMD EPYC 7713P, (2.7 MHz) takes about 5 min per participant, resulting in approximately 100 days of total compute time on our CPU cluster to process the entire cohort.

#### Validation

Model performance was evaluated on a held-out test set of 20 orbits (10 participants) excluded from training. To quantify the spatial overlap between automated and expert manual segmentations, the Dice Similarity Coefficient (DSC) was calculated. To evaluate the measurement agreement between the automated deep learning pipeline and manual expert segmentations in the held-out validation set, we calculated Intraclass Correlation Coefficients (ICC, two-way mixed-effects model for absolute agreement) and performed Bland-Altman analyses for key structures (total non-bony orbit, vitreous, anterior chamber, total optic nerve, combined extraocular muscles, and lens).

### Image post-processing and boundary refinement

All post-processing and subsequent morphometric analyses were performed using custom scripts in MATLAB 2021a (The MathWorks, Natick, MA, USA). Anatomical segmentations from the deep learning model were provided as an index (label) image, where each voxel was assigned to a specific anatomical structure.

#### Coordinate normalisation, midline alignment, and separation of left and right structures

To establish a consistent left–right reference frame, the center of mass of the optic chiasm segmentation was computed along the left–right (X) axis. All coordinates were shifted such that this center coincided with X = 0. For each segmentation label, connected components were identified to avoid merging contralateral structures across the midline. Components were assigned to the left or right side based on the mean X-coordinate of their voxels. If more than two connected components were detected for a single structure, the two largest were assigned bilaterally, and remaining components were quantified as “residual segmentation volume” (used subsequently for quality control).

#### Refinement of optic nerve and orbital boundaries

Alpha-shape reconstruction (*alphaShape* in MATLAB) was applied to the optic nerve segmentations to close gaps and fill internal cavities. Voxels enclosed by the alpha shape but not originally labelled were reassigned to the optic nerve class. To prevent the erroneous inclusion of optic nerve voxels outside the orbit, convex hulls of the orbital cavity were computed. Optic nerve voxels falling outside this hull were reassigned to surrounding tissue. Similarly, the nerve was anatomically bisected into “intraorbital” (within the orbit) and “intracranial” (posterior to the orbital apex/outside the orbit) segments.

### Morphometric analysis

Volumes were computed for all segmented structures based on voxel counts. The combined extraocular muscle volume was calculated as the sum of the four recti and two oblique muscles per orbit. Binary masks were defined for the lens and vitreous body on each side, and their geometric centers were calculated as the mean voxel coordinates of their respective masks.

#### Ocular axis estimation and eyeball dimensions

The principal (anteroposterior) ocular axis was defined as the normalized vector connecting the geometric center of the vitreous body to the geometric center of the lens. *Axial length* was calculated along this vitreous–lens axis, representing the distance between the anterior surface of the anterior chamber to the posterior surface of the vitreous. *Vertical and horizontal diameters* were calculated along the superior–inferior and horizontal directions, orthogonal to the principal ocular axis.

#### Orbital geometry and proptosis

The *orbital rim angle* was determined by computing the centroids of the lateral and medial orbital rim segmentations; vectors were constructed from the orbital apex to each rim centroid, and the enclosed angle was calculated using vector dot products. *Interzygomatic distance* was defined as the distance between the centroids of the left and right lateral orbital rims. *Eyeball proptosis* was measured as the perpendicular distance from the anterior-most point of the eyeball to the interzygomatic line.

#### Morphometry of elongated structures

For extraocular muscles and optic nerve segments, the largest connected component per side was retained. Segmentations were slightly dilated to ensure topological continuity, after which a 3D skeleton was extracted using a centerline tracing algorithm. From the ordered skeleton points, we computed the maximum diameter (reported as “length” in this manuscript), defined as the largest pairwise distance between voxels within the structure. Path length (cumulative Euclidean distance along the skeleton) and curvature (summed second-order finite difference along the centerline) were also computed.

### Quality control and data curation

A multilevel quality control process was employed to ensure the integrity of the morphometric data. First, automated segmentations from the training phase underwent visual inspection by three expert raters to confirm anatomical plausibility, identify systematic model failures, and manually correct errors.

Second, after the automated segmentation of the entire cohort (30,427 MRIs available after excluding 441 cases with missing data), we applied a statistical procedure to detect and remove multivariate outliers corresponding to segmentation failures. This analysis was performed on a vector of key morphometric parameters derived exclusively from the right eye, owing to the strong inter-ocular correlation for all major parameters and to prevent multicollinearity in subsequent models. We employed the robust Mahalanobis distance^22^, calculated using the Minimum Covariance Determinant (MCD) method, to measure how much each participant’s orbital morphometry profile deviated from the population distribution. The MCD estimator is robust to outliers and provides a stable estimate of central tendency.

To determine the appropriate exclusion threshold, we conducted a sensitivity analysis comparing a standard statistical cutoff based on the chi-square distribution (*p* < 0.001, α = 0.05) against a non-parametric interquartile range (IQR) approach flagging values > 1.5 × IQR. Two experts performed a manual review of segmentations flagged by both approaches, as well as non-outliers, using a stepwise approach with a 0.1 IQR difference in Mahalanobis distance. Visual inspection confirmed the IQR-based threshold was superior: it effectively captured participants with clear data quality issues—such as severe motion artifacts, corrupted image files, gross segmentation errors due to dental implants, or anatomies incompatible with the model (e.g., post-enucleation)—while the chi-square method was less specific. Consequently, the > 1.5 × IQR criterion was adopted for the final exclusion of participants from the normative analysis.

### Statistical analysis

All analyses were sex-stratified. For analyses of monocular parameters (e.g. lens, vitreous), only the right eye was analyzed. Descriptive statistics for baseline demographics and derived morphometric parameters are reported as mean [SD]. Differences between men and women were quantified by t-test, with p-values < 0.05 considered to denote statistical significance. Correlation with age was assessed by Spearman’s ρ with bootstrapped 95% confidence intervals (CI). Reference curves were derived by generalized additive regression and quantile regression with a cubic spline basis, accounting for variation of the conditional distribution of morphometric parameters by age. Mean, 5th, and 95th percentiles of the distribution were modelled and are provided as reference intervals in 5-year age bins. Statistical analyses were performed using R (Version 4.5.2, Foundation for Statistical Computing, Vienna, Austria) and GraphPad Prism (version 10.6.0; GraphPad Software, Boston, Massachusetts, USA).

## Results

### Study population and data curation

Baseline demographic and anthropometric characteristics of the included cohort are detailed in Table 1. A multilevel quality control procedure was applied to the morphometric data (see methods). From the initial 30,868 processed MRIs, 805 (2.6%) were excluded as statistical outliers. Manual review confirmed that these cases typically had severe motion artifacts, artifacts due to dental implants, or significant anatomical variations (e.g. prior surgical intervention). Additionally, 843 cases (2.7%) with low to absent lens volumes (threshold, ≤ 0.06 cm³) were excluded as aphakic or pseudophakic individuals, and for a further 441 cases (1.4%), no MRI images of the orbit were available. In total, 2,089 cases (6.8%) were excluded, resulting in a final dataset of 28,779 participants for statistical analysis (mean [SD] age, 48.1 [12.2] years; 44.1% [*n* = 12692] female; Supplementary Fig. S1A).

**Table 1:** Baseline demographic and anthropometric characteristics of the included study cohort.

Parameter	Available *N*	Overall	Men	Women	*p*-value
		28,779	16,095 (55.9%)	12,684 (44.1%)	
Age [years]	28,779	48.1 (12.2)	47.7 (12.3)	48.6 (12.0)	< 0.001
Height [cm]	28,752	173.1 (9.5)	179.0 (7.0)	165.6 (6.5)	< 0.001
Weight [kg]	28,752	79.6 (16.2)	86.3 (14.2)	71.1 (14.6)	< 0.001
BMI [kg/m2]	28,752	26.5 (4.7)	26.9 (4.1)	25.9 (5.3)	< 0.001
Education level	26,076				< 0.001
Low		457 (1.8)	201 (1.4)	256 (2.2)	
Medium		10,171 (39.0)	5,016 (34.4)	5,155 (44.9)	
High		15,448 (59.2)	9,379 (64.3)	6,069 (52.9)	
Imaging site	28,779				< 0.001
Augsburg		5,845 (20.3)	3,261 (20.3)	2,584 (20.4)	
Mannheim		5,476 (19.0)	3,163 (19.7)	2,313 (18.2)	
Essen		5,547 (19.3)	3,171 (19.7)	2,376 (18.7)	
Berlin		5,598 (19.5)	3,183 (19.8)	2,415 (19.0)	
Neubrandenburg		6,313 (21.9)	3,317 (20.6)	2,996 (23.6)	

Data are presented for the 28,779 participants from the German National Cohort (NAKO) included in the final analysis after quality control. Continuous variables are reported as mean (standard deviation, SD), and categorical variables are reported as count (percentage, %). BMI = Body Mass Index.

### Deep learning model performance and validation

**Fig. 2:**
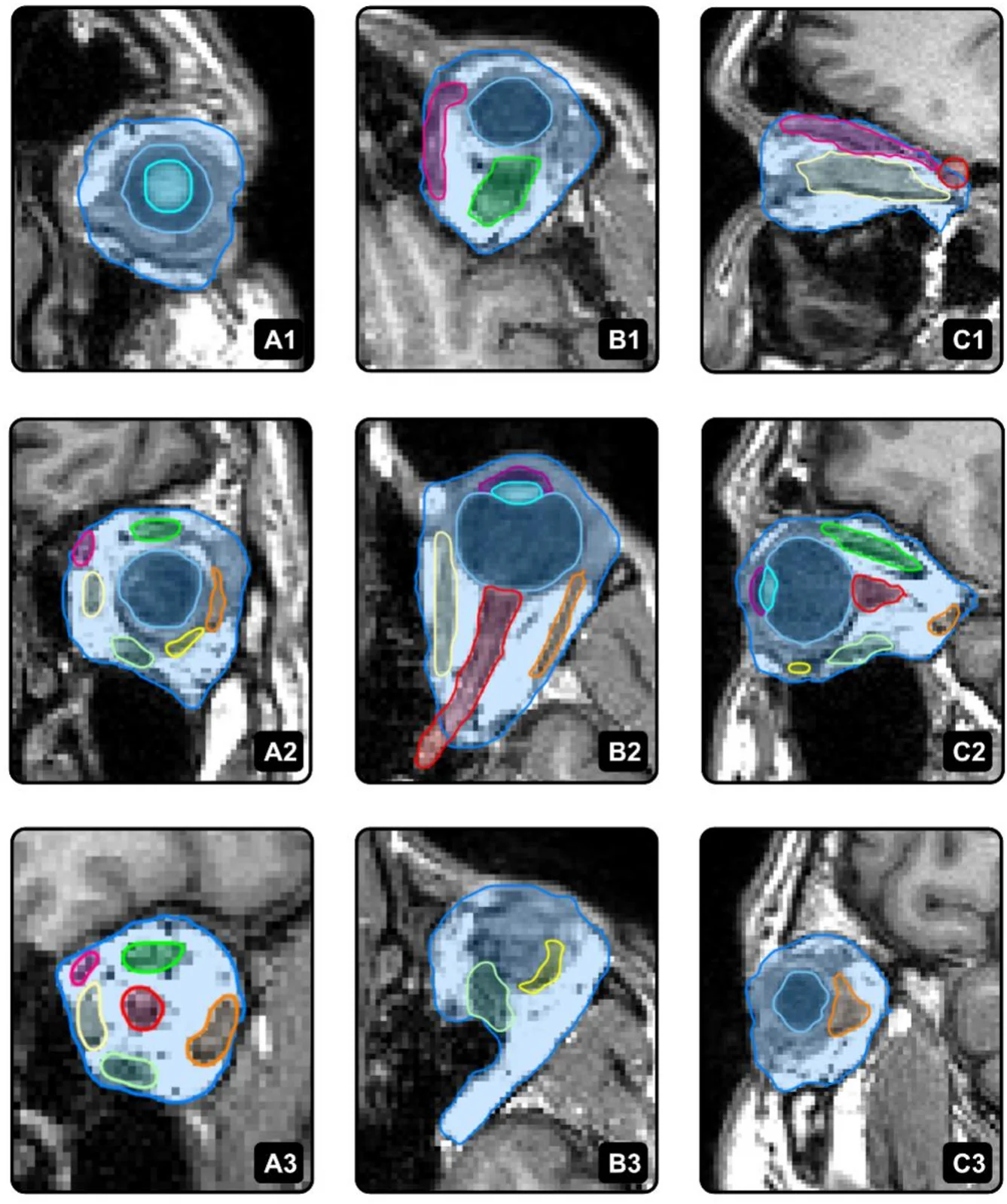
Representative example of manual ground truth segmentations. Manual segmentations are displayed as color overlays on T_1_-weighted MRI images to illustrate the ground truth data used for model training. Images are shown in three orthogonal planes at different anatomical slice levels: (A) coronal, (B) transverse, and (C) sagittal. Color key:  lens  anterior chamber  vitreous  remaining orbital non-bony tissue  superior rectus muscle  inferior rectus muscle  medial rectus muscle  lateral rectus muscle  superior oblique muscle  inferior oblique muscle  optic nerve.

**Fig. 3:**
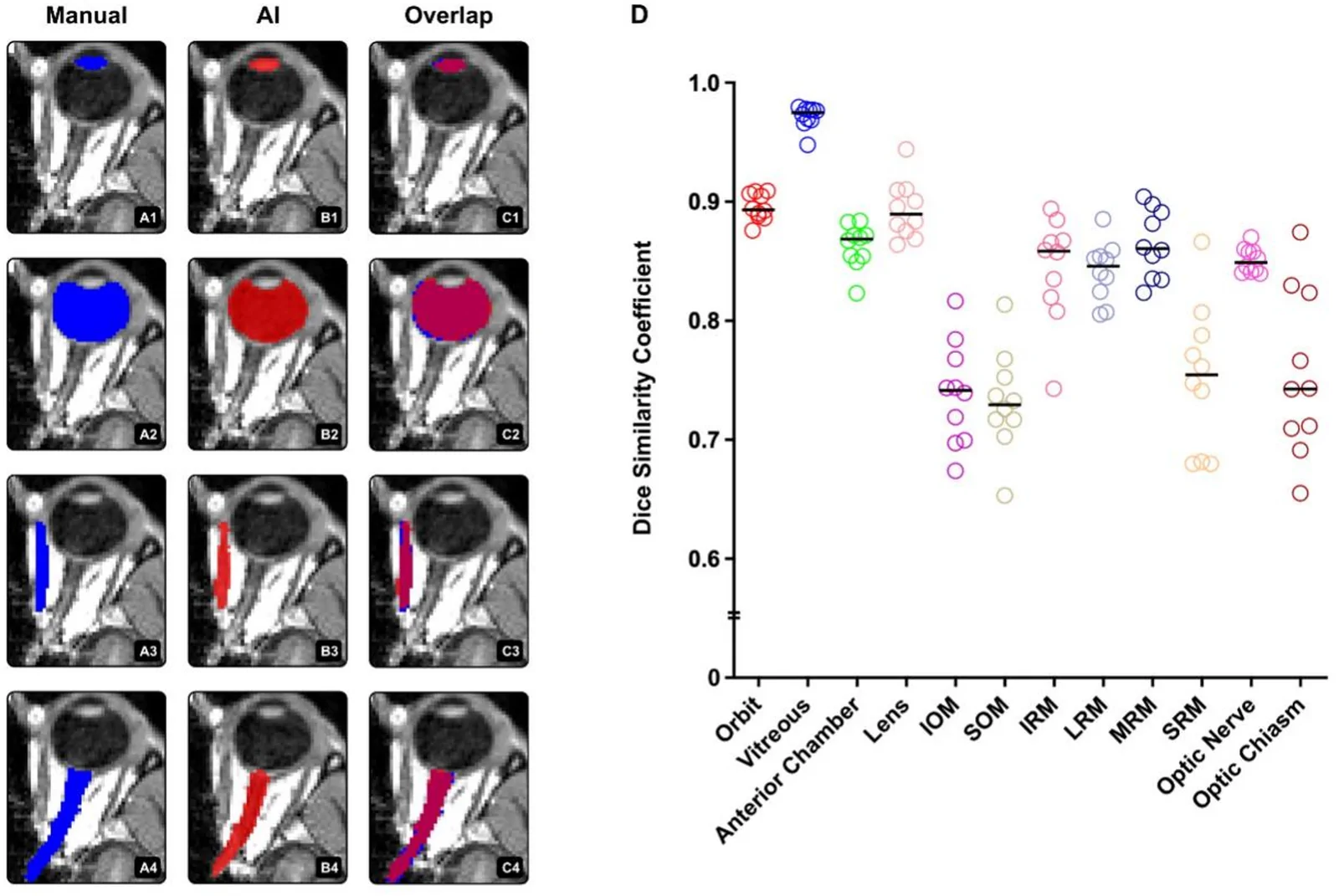
Validation of automated segmentation performance against manual ground truth. (**A-C**) Qualitative comparison of manual expert segmentations (blue, first column, A1-A4) and automated segmentations (red, second column, B1-B4) on the held-out test set. The spatial overlap is shown in purple (third column, C1-C4). Examples are provided for four representative structures: lens (row 1), vitreous (row 2), medial rectus muscle (row 3), and optic nerve (row 4), demonstrating a high degree of concordance. (**D**) Quantitative performance evaluation using the Dice Similarity Coefficient (DSC). The box plot shows the distribution of DSC scores for all analyzed structures, calculated on the held-out test set of 20 orbits (10 participants). The central line indicates the median. SRM: superior rectus muscle, IRM: inferior rectus muscle, MRM: medial rectus muscle, LRM: lateral rectus muscle, SOM: superior oblique muscle, IOM: inferior oblique muscle.

The model was trained using an iterative, expert-in-the-loop consensus process on a total of 322 orbits (Supplementary Fig. S1B). The final model demonstrated high segmentation accuracy across all targeted orbital structures (see Fig. 1 for schematic representation of all morphometric structures, see Fig. 2 for representative manual segmentations) when validated against the held-out validation set of 20 orbits (10 participants) that were not used during training or refinement (Fig. 3).

Mean DSCs indicated excellent agreement with the manual expert segmentations for key structures: anterior chamber (0.86), vitreous (0.97), lens (0.89), and intra-orbital optic nerve (0.85). Acceptable performance was achieved for more challenging, thinner tissues, such as the superior (0.73) and inferior (0.74) oblique muscles. Dice scores for all quantified structures are summarized in Fig. 3D. A representative example of the automated segmentation compared to the manual ground truth is provided in Fig. 3A-C, illustrating the high level of agreement across different orbital structures. Regarding Intraclass Correlation Coefficients (ICC), the automated pipeline demonstrated excellent absolute agreement for major large structures yielding ICC values of 0.92 for the total non-bony orbit and 0.96 for the vitreous. For smaller and morphologically more complex intra- and retrobulbar structures, the model achieved moderate to good reliability, with ICC values of 0.66 for the anterior chamber, 0.64 for the total optic nerve, 0.61 for the combined extraocular muscles (EOM), and 0.56 for the lens.

Bland-Altman analyses (Supplementary Fig. S2) revealed a small but consistent systematic bias in boundary definition between the AI and human raters. When accounting for the respective total volumes, the automated pipeline demonstrated a systematic negative bias for most solid structures, including the lens (mean difference − 0.0219 cm³; ~12% relative to total volume), combined EOM (-0.3938 cm³; ~10%), total optic nerve (-0.0468 cm³; ~5%), and the total orbit (-1.3111 cm³; ~6%). Conversely, a slight systematic positive bias was observed for the vitreous body (+ 0.1225 cm³; ~2%). This indicates that, at the 1-mm voxel level, the algorithm is marginally but systematically less inclusive at the boundaries of solid tissues compared to the human reference.

Furthermore, the limits of agreement (LoA) were acceptable and absolute variances remained small (Supplementary Fig. S2). While the LoA were tight for larger structures relative to their total volume (~ 5% variance for vitreous and total non-bony orbit), the relative width of the LoA for smaller structures (such as the lens and anterior chamber) was wider (~ 20% variance), reflecting the inherent ambiguity when manually delineating fine tissue boundaries on 1-mm isotropic MRI.

### Reference orbital morphometric data

Tables 2 and 3 detail the 25 volumetric (*n* = 16 volumes and *n* = 9 lengths) and 9 geometric parameters recorded.

**Table 2:** Reference volumetric data for orbital structures.

Parameter	Overall	Men	Women	*p*-value
**Volumes [cm³]**				
Orbit (total non-bony)	34.4 (3.9)	36.6 (3.3)	31.7 (2.8)	< 0.001
Anterior Chamber	0.25 (0.05)	0.27 (0.04)	0.23 (0.05)	< 0.001
Lens	0.17 (0.03)	0.18 (0.03)	0.17 (0.03)	< 0.001
Vitreous	6.3 (0.8)	6.5 (0.7)	6.1 (0.7)	< 0.001
Total eyeball	6.8 (0.8)	7.0 (0.8)	6.5 (0.7)	< 0.001
Optic nerve, intraorbital	0.72 (0.14)	0.77 (0.13)	0.65 (0.12)	< 0.001
Optic nerve, intracranial	0.26 (0.05)	0.28 (0.05	0.24 (0.04)	< 0.001
Optic nerve, total	0.98 (0.16)	1.05 (0.15)	0.90 (0.13)	< 0.001
Optic chiasm	0.26 (0.04)	0.27 (0.04)	0.24 (0.04)	< 0.001
Medial rectus	0.73 (0.12)	0.78 (0.11)	0.67 (0.09)	< 0.001
Lateral rectus	0.80 (0.14)	0.87 (0.12)	0.71 (0.10)	< 0.001
Inferior rectus	0.77 (0.13)	0.84 (0.11)	0.70 (0.10)	< 0.001
Superior rectus	0.96 (0.19)	1.03 (0.18)	0.86 (0.16)	< 0.001
Superior oblique	0.48 (0.08)	0.51 (0.07)	0.44 (0.06)	< 0.001
Inferior oblique	0.26 (0.05)	0.27 (0.05)	0.25 (0.05)	< 0.001
Extraocular muscles, combined	4.0 (0.6)	4.3 (0.5)	3.6 (0.4)	< 0.001
**Lengths (mm)**				
Optic nerve, intraorbital	29.6 (2.5)	30.7 (2.4)	28.3 (2.1)	< 0.001
Optic nerve, intracranial	20.1 (1.6)	20.7 (1.4)	19.4 (1.4)	< 0.001
Optic nerve, total	50.3 (3.4)	52.0 (2.9)	48.2 (2.6)	< 0.001
Medial rectus	32.8 (2.3)	33.8 (2.1)	31.5 (1.9)	< 0.001
Lateral rectus	37.4 (2.6)	38.7 (2.2)	35.7 (2.1)	< 0.001
Inferior rectus	33.5 (2.5)	34.5 (2.3)	32.2 (2.1)	< 0.001
Superior rectus	37.0 (2.2)	38.0 (1.9)	35.7 (1.8)	< 0.001
Superior oblique	36.2 (2.5)	37.1 (2.3)	35.0 (2.1)	< 0.001
Inferior oblique	19.1 (2.0)	19.3 (2.0)	18.7 (1.9)	< 0.001

Volumetric data derived from automated segmentations are presented as mean (SD) for the final cohort (*N* = 28,779), stratified by sex. All volumes are in cubic centimeters (cm³) and all lengths are in mm. For monocular structures, values from the right eye were used for analysis. The optic chiasm volume represents the total volume of the structure bilaterally. Statistical significance of the difference between sexes is provided.

**Table 3:** Reference geometric data for orbital structures.

Parameter	Overall	Men	Women	*p*-value
**Eyeball geometry [mm]**				
Axial length	23.5 (1.2)	23.8 (1.1)	23.1 (1.2)	< 0.001
Vertical diameter	23.8 (1.1)	24.0 (1.1)	23.5 (1.1)	< 0.001
Horizontal diameter	24.1 (1.1)	24.3 (1.0)	23.8 (1.0)	< 0.001
**Orbital geometry**				
Transverse diameter [mm]	40.3 (2.1)	40.9 (2.1)	39.6 (1.9)	< 0.001
Coronal diameter [mm]	48.8 (2.6)	49.9 (2.3)	47.3 (2.1)	< 0.001
Sagittal diameter [mm]	42.5 (1.9)	43.3 (1.7)	41.5 (1.6)	< 0.001
Orbital rim angle [°]	37.7 (1.5)	37.5 (1.5)	38.0 (1.4)	< 0.001
**Inter-orbital & relational [mm]**				
Interzygomatic distance [mm]	102.7 (4.5)	104.9 (3.9)	100.0 (3.7)	< 0.001
Eyeball proptosis [mm]	12.9 (1.8)	13.4 (1.8)	12.3 (1.7)	< 0.001

Geometric and linear measurement data are presented as mean (SD) for the final cohort (*N* = 28,779), stratified by sex. Lengths are in millimeters (mm) and angles are in degrees (°). For monocular parameters, values from the right side/eye were used for analysis. The interzygomatic distance is a binocular measurement. Statistical significance of the difference between sexes is provided.

### Volumetric findings (Table 2)

Mean total orbital volume (non-bony contents) was 34.4 [SD 3.9] cm³. The mean volumes of the major intraocular components were: vitreous, 6.3 [0.8] cm³; lens, 0.17 [0.03] cm³; anterior chamber, 0.25 [0.05] cm³. Key posterior structures included the intraorbital optic nerve volume, mean 0.72 [SD 0.14] cm³, intracranial optic nerve volume, 0.26 [0.05] cm³, and the combined volume of the six extraocular muscles (EOMs), 4.0 [0.6] cm³.

### Geometric findings (Table 3)

The mean axial length of the eyeball, defined as the distance from the anterior surface of the anterior chamber to the posterior margin of the vitreous, was 23.5 [SD 1.2] mm. Vertical and horizontal diameters of the eyeball were 23.8 [1.1] mm and 24.1 [1.1] mm, respectively. Geometric analysis further yielded a mean eyeball proptosis of 12.9 [1.8] mm, a mean orbital rim angle of 37.7 [1.5] degrees, and a mean interzygomatic distance of 102.7 [4.5] mm.

### Associations with sex

Systematic sexual dimorphism was observed across all 34 parameters, with males consistently exhibiting significantly larger dimensions (Tables 2 and 3; *p* < 0.001). For instance, the mean total orbital volume was significantly greater in males (36.6 [3.3] cm³) than females (31.7 [2.8] cm³). Similarly, mean axial length was 23.8 [1.1] mm in males versus 23.1 [1.2] in females.

### Associations with age

**Fig. 4:**
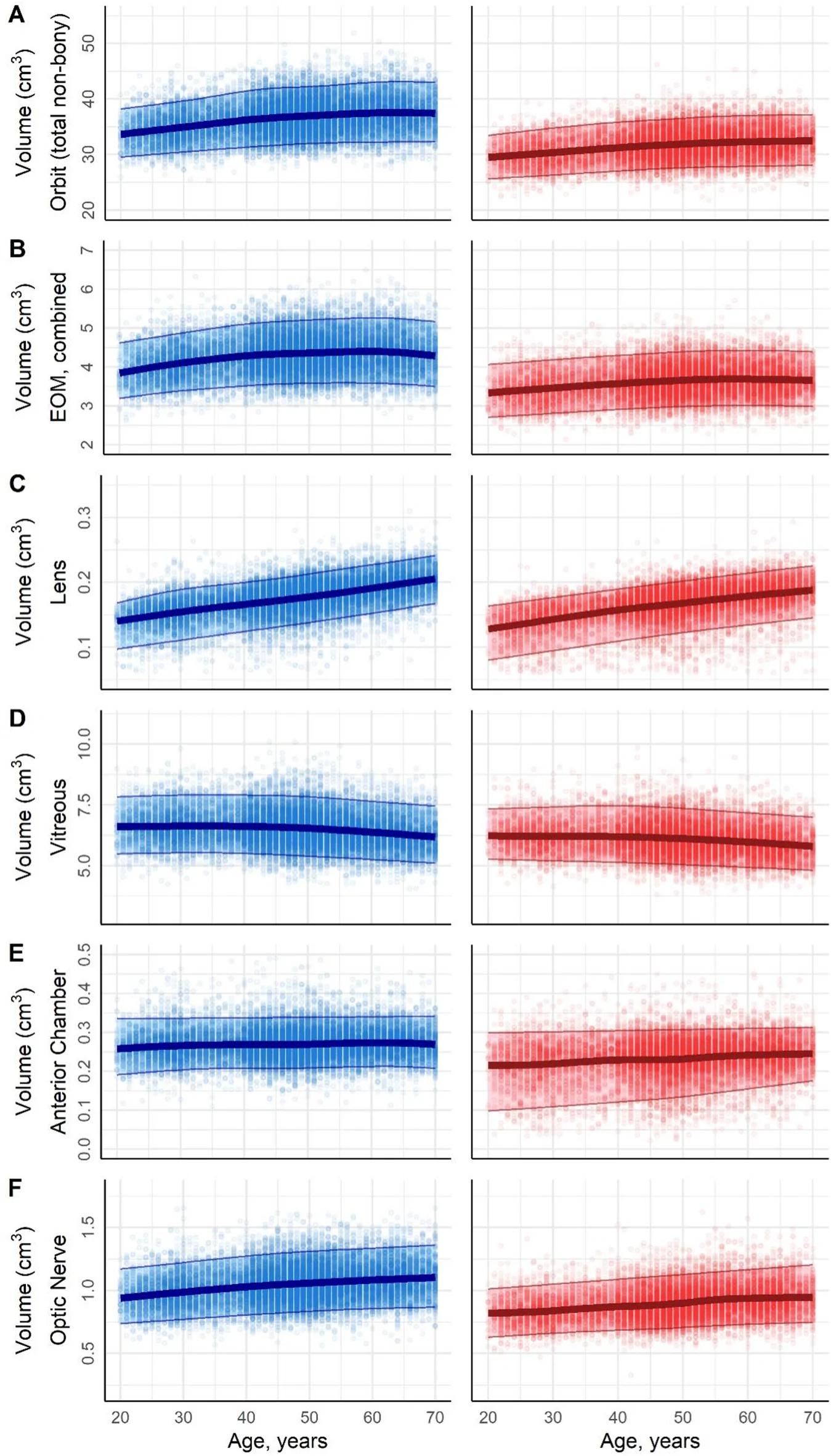
Age-stratified percentile curves (5th, 50th, 95th) for morphometric parameters. On the x-axis: Age in years. On the y-axis: total orbital volume (**A**), combined extraocular muscle (EOM) volume (**B**), lens volume (**C**), vitreous volume (**D**), anterior chamber volume (**E**), total optic nerve volume (**F**), all in cm^3^, for men (blue) and women (red). Mean, 5th and 95th percentiles of the distribution were derived by generalized additive quantile regression with cubic spline basis, allowing the conditional distribution of each morphometric parameter to vary with age.

Normative reference data according to age are given in Fig. 4; Table 4. Age-stratified percentile curves (Fig. 4) revealed distinct morphometric patterns across the observed lifespan. Total lens volume demonstrated continuous pronounced growth in both sexes (Spearmen’s ρ = 0.57, 95% CI [0.55, 0.58] in men; ρ = 0.51 [0.50, 0.53] in women). Mean (50th percentile) lens volume in males increased by approximately 40%, from 0.14 cm³ in the 20–25 age group to 0.20 cm³ in those > 65 years. Modest age-related increases were observed for total orbital volume (ρ = 0.28 [0.26, 0.29] in men; ρ = 0.24 [0.22, 0.26] in women), optic nerve volume (ρ = 0.25 [0.23, 0.26] in men; ρ = 0.27 [0.26, 0.29] in women), and combined EOM volumes (ρ = 0.19 [0.17, 0.21] in men; ρ = 0.17 [0.16, 0.19] in women). Anterior chamber volumes remained stable (ρ = 0.07 [0.06, 0.09] in men, ρ = 0.14 [0.13, 0.16] in women), while vitreous volume showed a slight age-related decline (ρ=-0.16 [-0.17, -0.14] in men, ρ=-0.16 [-0.18, -0.14] in women).

**Table 4:** Reference data by age.

		Age, years									
Volume, cm^3^		[20,25)	[25,30)	[30,35)	[35,40)	[40,45)	[45,50)	[50,55)	[55,60)	[60,65)	65+
Orbit (total non-bony)	Men	34.0 (29.8, 38.6)	34.6 (30.2, 39.2)	35.2 (30.6, 40.0)	35.9 (31.1, 40.9)	36.4 (31.5, 41.7)	36.8 (31.8, 42.1)	37.1 (32.1, 42.3)	37.3 (32.2, 42.8)	37.5 (32.3, 43.1)	37.5 (32.3, 43.1)
	Women	29.7 (25.8, 33.8)	30.1 (26.1, 34.4)	30.5 (26.4, 35.0)	31.0 (26.8, 35.5)	31.4 (27.2, 36.0)	31.7 (27.5, 36.3)	32.0 (27.7, 36.7)	32.2 (27.8, 36.9)	32.3 (27.9, 37.1)	32.4 (28.0, 37.1)
Combined extraocular muscles	Men	3.9 (3.2, 4.7)	4.0 (3.3, 4.8)	4.1 (3.4, 4.9)	4.2 (3.5, 5.0)	4.3 (3.5, 5.1)	4.3 (3.6, 5.2)	4.4 (3.6, 5.2)	4.4 (3.6, 5.3)	4.4 (3.6, 5.3)	4.3 (3.5, 5.2)
	Women	3.4 (2.7, 4.1)	3.4 (2.8, 4.2)	3.5 (2.8, 4.2)	3.5 (2.9, 4.3)	3.6 (2.9, 4.3)	3.6 (3.0, 4.4)	3.7 (3.0, 4.4)	3.7 (3.0, 4.4)	3.7 (3.0, 4.4)	3.7 (3.0, 4.4)
Lens	Men	0.14 (0.1, 0.17)	0.15 (0.11, 0.18)	0.16 (0.11, 0.19)	0.16 (0.12, 0.20)	0.17 (0.13, 0.20)	0.17 (0.13, 0.21)	0.18 (0.14, 0.22)	0.19 (0.15, 0.22)	0.19 (0.16, 0.23)	0.20 (0.16, 0.24)
	Women	0.13 (0.08, 0.17)	0.14 (0.09, 0.17)	0.15 (0.10, 0.18)	0.15 (0.10, 0.19)	0.16 (0.11, 0.19)	0.17 (0.12, 0.20)	0.17 (0.12, 0.20)	0.18 (0.13, 0.21)	0.18 (0.14, 0.22)	0.19 (0.14, 0.22)
Vitreous	Men	6.6 (5.5, 7.9)	6.6 (5.5, 7.9)	6.6 (5.5, 7.9)	6.6 (5.5, 7.9)	6.6 (5.5, 7.9)	6.6 (5.4, 7.9)	6.5 (5.4, 7.8)	6.4 (5.3, 7.7)	6.3 (5.2, 7.6)	6.2 (5.2, 7.5)
	Women	6.2 (5.3, 7.4)	6.2 (5.2, 7.4)	6.2 (5.2, 7.4)	6.2 (5.2, 7.5)	6.2 (5.1, 7.5)	6.1 (5.1, 7.4)	6.1 (5.0, 7.3)	6.0 (5.0, 7.2)	5.9 (4.9, 7.1)	5.9 (4.9, 7.1)
Anterior chamber	Men	0.26 (0.19, 0.34)	0.26 (0.20, 0.34)	0.27 (0.21, 0.34)	0.27 (0.21, 0.34)	0.27 (0.21, 0.34)	0.27 (0.21, 0.34)	0.27 (0.21, 0.34)	0.27 (0.21, 0.34)	0.27 (0.21, 0.34)	0.27 (0.21, 0.34)
	Women	0.22 (0.10, 0.30)	0.22 (0.11, 0.30)	0.22 (0.11, 0.30)	0.23 (0.12, 0.30)	0.23 (0.12, 0.31)	0.23 (0.13, 0.31)	0.23 (0.14, 0.31)	0.24 (0.15, 0.31)	0.24 (0.16, 0.31)	0.24 (0.17, 0.31)
Total optic nerve	Men	0.95 (0.75, 1.18)	0.97 (0.76, 1.21)	1.00 (0.78, 1.23)	1.02 (0.80, 1.26)	1.04 (0.81, 1.28)	1.05 (0.83, 1.30)	1.06 (0.84, 1.32)	1.08 (0.85, 1.33)	1.09 (0.86, 1.34)	1.10 (0.86, 1.35)
	Women	0.82 (0.64, 1.02)	0.83 (0.65, 1.04)	0.85 (0.67, 1.06)	0.86 (0.68, 1.08)	0.88 (0.69, 1.10)	0.89 (0.70, 1.12)	0.91 (0.71, 1.13)	0.93 (0.72, 1.15)	0.94 (0.74, 1.17)	0.94 (0.74, 1.19)

Volumes of segmented ocular and orbital structures by age groups (in cm³). Data are represented as mean, 5th and 95th percentiles of the distribution.

## Discussion

In this study, we developed and validated a fully automated deep learning pipeline to analyze T_1_-weighted MRIs from over 30,000 NAKO participants, generating comprehensive, population-based reference data for 34 orbital parameters. This tool effectively overcomes the prohibitive labor and subjectivity of manual MRI segmentation.

The primary strength of our study is the unprecedented scale and standardization of the dataset. Previous MRI-based orbital morphometry studies were limited to much smaller, often clinical or single-center cohorts (largest study with *n* = 1,926 subjects) prone to selection bias and limited generalizability^23–25^. NAKO provides a prospective, population-based sample with a highly standardised MRI acquisition protocol across all imaging centres, ensuring data harmonization and statistical power^12^. Our automated pipeline makes this vast and valuable resource tractable, enabling orbital investigations at a large scale. High segmentation accuracy, with Dice Similarity Coefficients of 0.97 for the vitreous, 0.89 for the lens, and 0.85 for the optic nerve, confirms that our approach is a reliable, efficient substitute for manual expert delineation while being much less time consuming. For comparison, while the manual segmentation of the total orbit for ground truth generation required 8–12 h per orbit, the finalised automated pipeline processed a single participant (both orbits) in 5 min on a standard CPU. While the training set of 161 participants (322 orbits) may appear modest compared to typical 2D image classification datasets, it represents a large ground-truth library for 3D orbital morphometry, requiring approximately 3,000 expert hours to generate. The adequacy of this dataset size is driven by the patch-based nature of our 3D segmentation architecture, which extracts millions of training instances from these volumes. Furthermore, the strict standardization of the NAKO imaging protocol across identical 3-T scanners minimizes inter-scan variance, allowing the model to achieve robust intra-cohort generalization without the need for thousands of full-volume manual segmentations. Crucially, our volumetric validation using Intraclass Correlation Coefficients and Bland-Altman analyses demonstrated that while absolute agreement was moderate for the smallest structures, the model delivers consistent quantitative volume measurements. Bland-Altman plots revealed a slight, systematic negative volumetric bias for most solid structures (e.g., lens, EOM) alongside a minimal positive bias for the vitreous, indicating that the algorithm systematically applies a marginally less inclusive boundary definition than the human rater. While this boundary ambiguity leads to wider relative limits of agreement for tiny structures (reflecting the inherent ambiguity when manually delineating fine tissue boundaries on relatively thick (1 mm) isotropic MRI), the automated pipeline standardizes this boundary definition and eliminates intra- and inter-observer drift.

The validity of our automated measurements is supported by their consistency with established biological principles and alternative measurement techniques. For instance, our mean axial length of 23.5 mm is consistent with, though slightly shorter than, values reported in large population-based studies using optical biometry, like the Gutenberg Health Study^26^. This small, systematic difference is expected, as the T_1_-weighted images of the NAKO study enabled us to measure the distance between the anterior surface of the anterior chamber to the posterior margin of the vitreous, whereas optical biometry measures from the anterior surface of the cornea to the retinal pigment epithelium^27^. Furthermore, the detected lifelong growth of the crystalline lens and the systematic sexual dimorphism - with males exhibiting larger orbital dimensions - perfectly mirror established anthropometric data^24,25,28,29^.

Our reference data open numerous avenues for future clinical and epidemiological research: Quantifying EOM and orbital volumes facilitates research into thyroid eye disease, where EOM enlargement is a key diagnostic feature^30,31^. These data will help establish muscle-to-orbit reference ratios, improving diagnostic accuracy and enabling research into subclinical changes. Volumetric optic nerve assessment will enable investigations into neurodegenerative optic neuropathies like glaucoma^32,33^. Additionally, geometric parameters like axial length can now be correlated with anthropometric parameters like body height as well as social parameters such as education level, across the broader NAKO cohort.

Several limitations must be acknowledged. First, the MRI data were acquired using a brain imaging protocol with 1.0 mm isotropic resolution, which is lower than that of dedicated, high-resolution orbital MRI. While sufficient for major orbital structures, this resolution may limit the precision for small structures, such as the oblique muscles. Second, we lack gold-standard clinical ophthalmological measures (e.g. objective refraction, intraocular pressure) and physician-confirmed diagnoses. Reliance on self-reported diagnoses limits our ability to capture undiagnosed diseases or precisely analyze refractive errors. Third, the NAKO cohort’s age range (20–74 years) means our reference data cannot be extrapolated to pediatric or highly elderly populations, where distinct anatomical characteristics are expected. Fourth, this study is cross-sectional, allowing us to identify associations (e.g. with sex) but not to infer causality or track longitudinal changes within individuals. Finally, there are specific limitations regarding the generalizability of the proposed deep learning model. The algorithm was trained exclusively on T1-weighted MPRAGE sequences acquired on identical 3-T Siemens scanners within a general population cohort. Consequently, its performance on images from different scanner vendors, lower or higher magnetic field strengths (e.g., 0.55 T, 1.5 T, 5.0 T, 7.0 T), alternative MRI contrast mechanisms (e.g., T2-weighted imaging or diffusion weighted imaging), or in patients with severe orbit-distorting pathologies (such as ocular and orbital tumors or significant trauma) remains unproven. Applying this specific model to diverse external clinical datasets would likely require transfer learning or dataset-specific fine-tuning.

In summary, we have demonstrated the feasibility and accuracy of a deep learning-based pipeline for automated orbital morphometry in a large-scale population study. This work provides an extensive, robust set of age- and sex-stratified reference data for the German population. Moving forward, this foundational resource will enable researchers to efficiently investigate the complex relationships between orbital anatomy, systemic diseases, and environmental exposures captured within the NAKO dataset.

## Supplementary Information

Below is the link to the electronic supplementary material.


Supplementary Material 1



Supplementary Material 2


## Data Availability

Due to strict data protection regulations and the informed consent framework of the NAKO study, the data used in this study cannot be made publicly available. However, access to and use of NAKO (German National Cohort) data and biospecimens can be obtained via an electronic application portal (https://transfer.nako.de/transfer/index). To facilitate reproducibility, the trained deep learning model has been deposited in a public repository and can be accessed at (https://bitbucket.org/reisert/eye_model/src/main/). The custom MATLAB scripts used for post-processing and morphometric analysis are available upon reasonable request.
