# Supplementary material for: Volumetric reference data of the orbit: a deep learning MRI analysis in the German national cohort
Source: Sci Rep. 2026 Aug 29;16:27113. doi: 10.1038/s41598-026-68393-x (PMC13526028; doi:10.1038/s41598-026-68393-x)

Figure S1. Overview of the data curation and iterative deep learning training pipeline

(A) Flowchart illustrating the data curation process, from the initial NAKO MRI cohort of 30,868 participants to the final analysis set of 28,779 participants after a multi-level quality control process.
(B) The "expert-in-the-loop" training workflow. An initial model was trained on a small set of manual segmentations. This model then generated pre-segmentations, which were reviewed and corrected by a consensus of expert raters. This refined dataset was used to retrain the model iteratively, progressively improving its accuracy. The example shows an initial automated segmentation (bottom middle) and the subsequent expert-corrected version (bottom right), highlighting a refinement of the anterior chamber and lens boundaries.

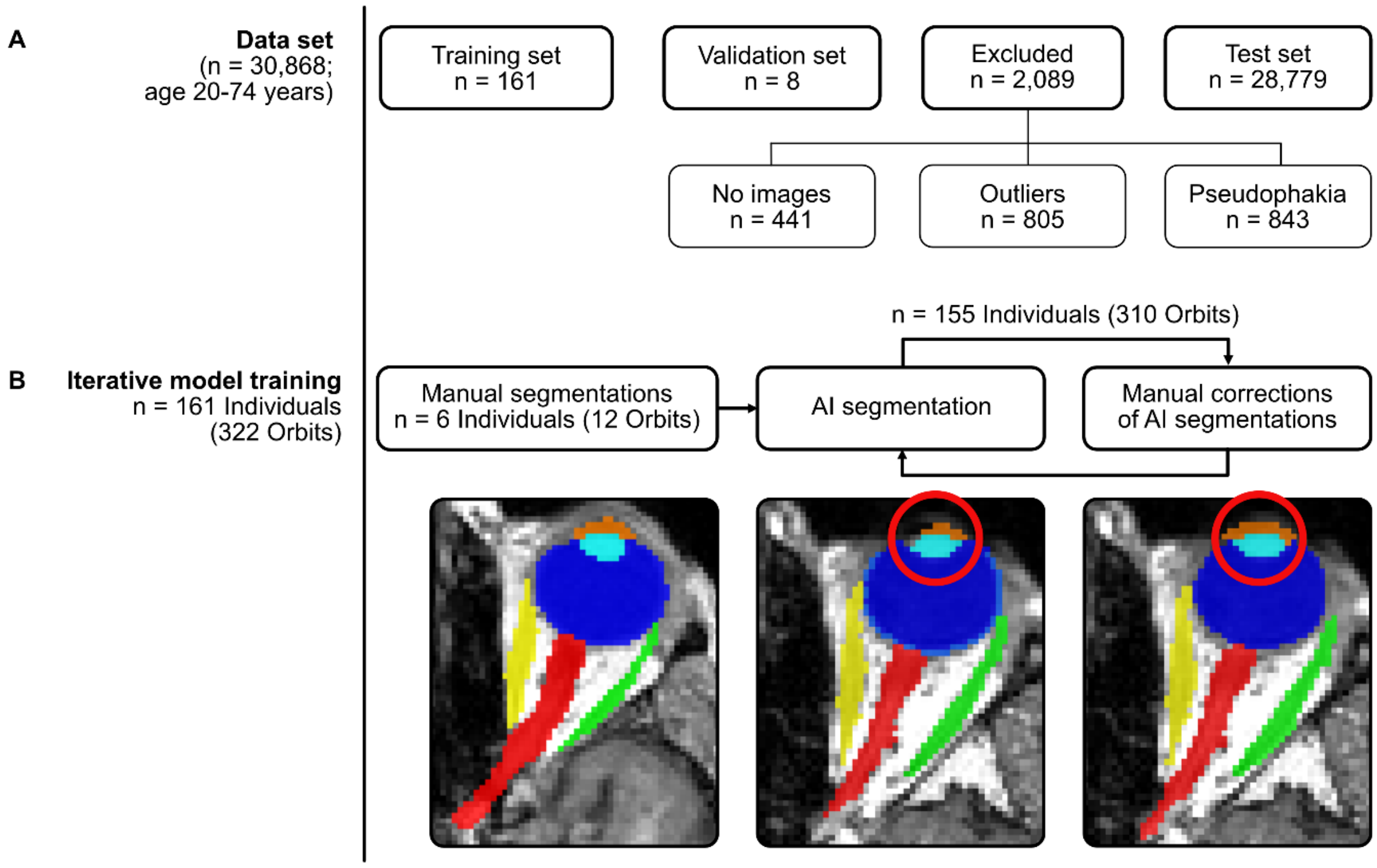

Supplement: Supplementary file 1 [file 41598_2026_68393_MOESM1_ESM.pdf]
