# Supplementary material for: Volumetric reference data of the orbit: a deep learning MRI analysis in the German national cohort
Source: Sci Rep. 2026 Aug 29;16:27113. doi: 10.1038/s41598-026-68393-x (PMC13526028; doi:10.1038/s41598-026-68393-x)

Figure S2. Bland-Altman plots comparing automated deep learning segmentations to manual expert ground truth

Bland-Altman plots demonstrate the volumetric agreement between manual and automated measurements (in cm³) for six key orbital structures: total non-bony orbit, anterior chamber, lens, vitreous, total optic nerve, and combined extraocular muscles (EOM). Data are derived from the held-out test set (n = 20 orbits from 10 participants). The x-axis represents the mean volume of the two measurement methods, while the y-axis represents the absolute difference (Automated AI volume – Manual expert volume). The solid black line indicates perfect agreement (zero difference). The middle dashed line represents the mean difference (systematic bias) between the methods, with the grey shaded region illustrating the 95% confidence interval of this mean difference. The upper and lower dotted lines represent the 95% limits of agreement (mean difference ± 1.96 standard deviations). Consistent with partial volume effects at 1-mm isotropic MRI resolution, the AI demonstrates a slight systematic negative bias for solid tissue structures (e.g., lens, EOM, orbit) and a corresponding minimal positive bias for the vitreous fluid, indicating a marginally less inclusive boundary definition by the automated algorithm compared to the human rater.

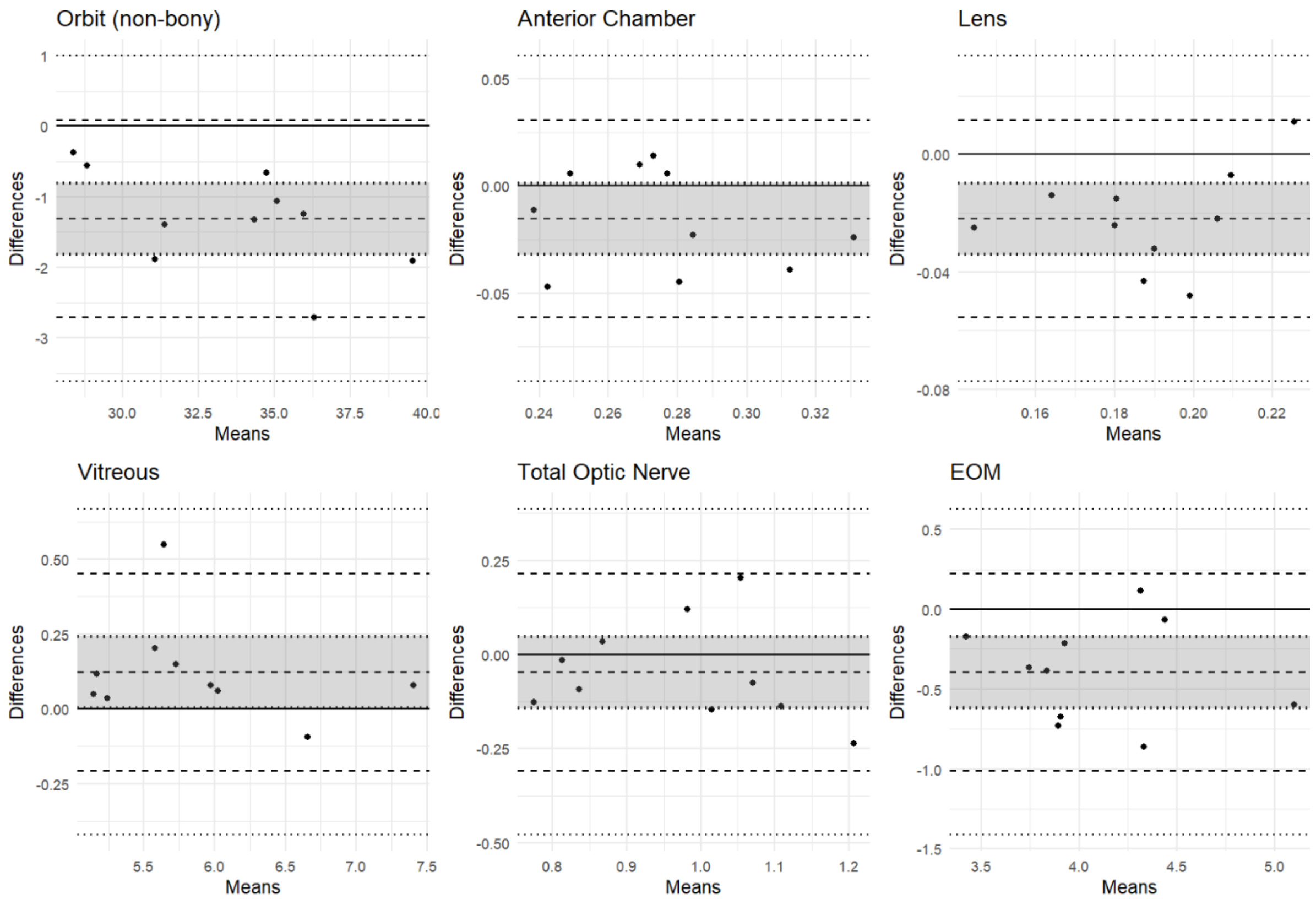

Supplement: Supplementary file 2 [file 41598_2026_68393_MOESM2_ESM.pdf]
